# Prediction of low Apgar score at five minutes following labor induction intervention in vaginal deliveries: machine learning approach for imbalanced data at a tertiary hospital in North Tanzania

**DOI:** 10.1186/s12884-022-04534-0

**Published:** 2022-04-01

**Authors:** Clifford Silver Tarimo, Soumitra S. Bhuyan, Yizhen Zhao, Weicun Ren, Akram Mohammed, Quanman Li, Marilyn Gardner, Michael Johnson Mahande, Yuhui Wang, Jian Wu

**Affiliations:** 1 Department of Epidemiology and Health Statistics, College of Public Health, Zhengzhou University, 100 Kexue Avenue, Zhengzhou, 450001 Henan China; 2 Department of Science and Laboratory Technology, Dar es Salaam Institute of Technology, P.O. Box 2958, Dar es Salaam, Tanzania; 3 Rutgers University-New Brunswick, Edward J. Bloustein, School of Planning and Public Policy, New Brunswick, USA; 4 Luoyang Orthopedic Traumatological Hospital of Henan Province, Luoyang, China; 5 College of Sanquan, Xinxiang Medical University, Xinxiang, People’s Republic of China; 6 Center for Biomedical Informatics, University of Tennessee Health Science Center, Memphis, TN USA; 7 Department of Public Health, Western Kentucky University, 1906 College Heights Blvd, Bowling Green, KY 42101 USA; 8 Institute of Public Health, Kilimanjaro Christian Medical University College, P.O. Box 2240, Moshi, Tanzania; 9 Centre for Financial and Corporate Integrity, Coventry University, Coventry, UK; 10 Henan Province Engineering Research Center of Health Economics & Health Technology Assessment, Henan Province, China

**Keywords:** Low five-minute Apgar score, Successful labor induction, Machine learning, Imbalanced data, North-Tanzania

## Abstract

**Background:**

Prediction of low Apgar score for vaginal deliveries following labor induction intervention is critical for improving neonatal health outcomes. We set out to investigate important attributes and train popular machine learning (ML) algorithms to correctly classify neonates with a low Apgar scores from an imbalanced learning perspective.

**Methods:**

We analyzed 7716 induced vaginal deliveries from the electronic birth registry of the Kilimanjaro Christian Medical Centre (KCMC). 733 (9.5%) of which constituted of low (< 7) Apgar score neonates. The ‘extra-tree classifier’ was used to assess features’ importance. We used Area Under Curve (AUC), recall, precision, F-score, Matthews Correlation Coefficient (MCC), balanced accuracy (BA), bookmaker informedness (BM), and markedness (MK) to evaluate the performance of the selected six (6) machine learning classifiers. To address class imbalances, we examined three widely used resampling techniques: the Synthetic Minority Oversampling Technique (SMOTE) and Random Oversampling Examples (ROS) and Random undersampling techniques (RUS). We applied Decision Curve Analysis (DCA) to evaluate the net benefit of the selected classifiers.

**Results:**

Birth weight, maternal age, and gestational age were found to be important predictors for the low Apgar score following induced vaginal delivery. SMOTE, ROS and and RUS techniques were more effective at improving “recalls” among other metrics in all the models under investigation. A slight improvement was observed in the F1 score, BA, and BM. DCA revealed potential benefits of applying Boosting method for predicting low Apgar scores among the tested models.

**Conclusion:**

There is an opportunity for more algorithms to be tested to come up with theoretical guidance on more effective rebalancing techniques suitable for this particular imbalanced ratio. Future research should prioritize a debate on which performance indicators to look up to when dealing with imbalanced or skewed data.

**Supplementary Information:**

The online version contains supplementary material available at 10.1186/s12884-022-04534-0.

## Background

Labor induction (IOL) is a procedure in which a physician or midwife uses methods to help a pregnant woman go into labor [1, 2]. The factors for IOL procedure may be classified as maternal, fetal, social [3]. IOL rates have continued to rise over the past few decades, owing to a growing focus on reducing perinatal morbidity and mortality [4, 5]. IOL prevalence varies greatly between countries and regions globally but developed countries have reported higher rates than developing ones [6]. In the UK and the US, IOL accounts for about 20% of deliveries, but rates have been steadily increasing over the last decade [7]. The IOL rate in Africa is currently at 4.4%, confirming the region’s lowest rates for this important intervention. A successful IOL should lead to vaginal delivery [8]. Recently, advances in techniques of obstetric and fetal monitoring, most induced pregnancies have favorable outcomes, however adverse health outcomes leading to low Apgar score in neonates still exist [9]. Early detection of a low Apgar score helps ensure survival of the newborn [10]. However, the imbalanced class distribution of pregnancy outcomes, along with the complexity of assessment, lead to bare investigations on the predictions of severely low prevalent outcomes including Apgar score following successful IOL intervention [11]. The Apgar score is a standardized and well accepted method to measure and assess newborn’s health condition immediately after delivery [12]. Though the use of Apgar score tool to predict neonatal outcomes has been discouraged in some studies [13], it has been widely used to provides an accepted and convenient method for reporting the status of the newborn infant immediately after birth and the response to resuscitation in Sub-Saharan Africa including Tanzania. Five components assessed include heart rate, respiration, reflexes, muscle tone, and color. Each component is given a score of 0, 1, or 2 [14]. To reduce the ‘noise’ from the partially subjective nature of the scoring (eg, the “color” component), the pioneer of this system, Dr. Apgar, suggested categorizing the composite score as low (0–3), intermediate (4–6), and normal (7–10). However, data from a population-based study reported that Apgar scores of 7, 8, and 9 versus 10 were also associated with higher neonatal mortality and morbidity [15]. Numerous factors, including gestational age, maternal medication and anesthesia during pregnancy and labor, congenital anomalies and interobserver variability, may affect the Apgar score [16]. Although Apgar score may not be appropriate for predicting individual’s neurological outcomes [16], multiple studies have looked into the relationship between Apgar score value and death or neurologic impairment in the newborn at the population level [17]. Antenatal and peripartum adversities associated with low Apgar scores have been implicated in neonatal brain injury, which in turn may lead to neurodevelopmental disability [18]. The first and the five-minute Apgar scores have been identified as independent predictors of neonatal morbidity and mortality; however, the five-minute score is considered to be a more accurate predictor of outcome regardless of birth weight [19, 20]. A low five-minute score is linked to a higher risk of neurological disorders such as cerebral palsy, epilepsy, cognitive impairment, and hyperactivity disorder later in life [21]. Studies have shown increased risk of long term neurological disability that persist into young adulthood with intermediate Apgar scores at 5 minutes [22].

Despite a wealth of information on the relationship between low Apgar score and short- and long-term adverse health outcomes for newborns, predictive modeling studies are still scarce. As medicine undergoes an electronic revolution, data becomes more accessible, laying the groundwork for computer-mediated personalized medicine. A substantial number of machine learning (ML) methods for automatic detection of pregnancy outcomes have been developed, and most of them reported high classification accuracy [23]. However, class imbalance problem has been reported to impart the predictive efficiency of these models [24]. In this case, conventional machine learning algorithms are likely to be overwhelmed by the majority class and neglect the minority class, as traditional classifiers strive for accuracy over a broad range of instances [25]. However, no study has been conducted on the evaluate models’ performance the classification of low versus normal five-minute Apgar score in normal vaginal delivery following IOL intervention. In the current study, we hypothesized that machine learning models can perform well in the presence of class imbalance following the application of resampling techniques. Effective modeling for a low-Apgar-score newborn following a successful labor induction intervention would aid in ensuring prompt clinical management and resource allocation and hence improvement in pregnancy outcomes.

## Methods

### Study setting and data collection

Electronic birth registry records were retrospectively extracted from the department of Obstetrics and Gynecology of the Kilimanjaro Christian Medical Center (KCMC) from the year 2000 to 2015. This facility serves residents of Kilimanjaro and the surrounding regions in northern Tanzania. Since the year 2000, information on pregnancy, delivery, and newborns has been recorded in a specific database. After each uncomplicated delivery, trained nurses conduct personal interviews every day, or every 3 days for complicated deliveries. Interviews were carried out using structured questionnaire. The database for hospital birth registration records contains sociodemographic information about mothers and information about their health before and after delivery. Clinical data coverage includes parity, labor induction, referral status, IOL indications, induction methods used, pregnancy history and pregnancy outcome data including Apgar score at one and 5 minutes.

### The outcome variable & eligibility criteria

The outcome variable used in this study is the Apgar score at 5 minutes from induced vaginal deliveries. We selected the five-minute Apgar score as it provides information on how well the baby is functioning outside the womb and induced vaginal deliveries as it is the desired outcome for IOL. The scoring protocol used by midwives and clinicians for each component of the Apgar score is summarized in Table 1. The investigator summed up the five scores, then reclassified the sums as “low” if the score was < 7 and “normal” if the score was ≥7. The outcome variable was then encoded as binary input scaled [0, 1].

**Table 1:** Scoring guideline for Apgar score

Sign	0	1	2
Heart rate	Absent	< 100	≥100
Respiratory effort	Absent	Weak cry, hypoventilation	Good, crying
Reflex irritability	No response	Grimace	Cry or active withdrawal
Muscle tone	Limp	Some flexions of extremities	Active motion
Color	Blue, pale	Body pink, extremities blue	Completely pink

**Fig. 1:**
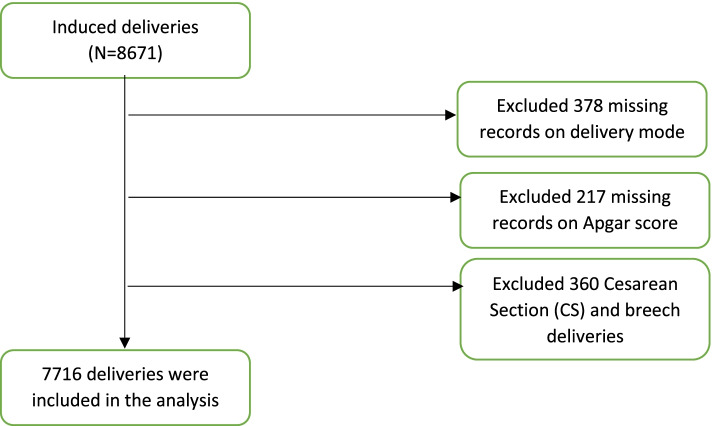
Consort diagram for participants recruitment

Eligibility criteria included normal delivery following IOL. Thus, we excluded cesarean sections and non-vertex presentation, as well as deliveries which had missing value on delivery mode or missing value on Apgar score. The training set had 5401 while the validation set contained 2315 deliveries (Fig. 1).

### Feature importance and variable correlation

Using Apgar score as dependent variable (coded 0 (≥7) and 1 (< 7)), we used “Extra-trees classifiers” using Scikit-learn toolkit in Python version 3.7.4, to determine covariates with the highest predictive power in comparison to others in a complete dataset. In these ensemble methods, each variable is ordered in descending order according to the Gini Importance of each feature and the top features are selected [26, 27]. In this method, we looked at the relative values of the computed importance whereby the higher the gini index value the more important is the variable. Additionally, we performed correlation analysis among the variable included in this dataset.

### Imbalanced learning model establishment

In conventional machine learning algorithms, dealing with imbalanced data is listed among the ten most challenging tasks in data mining research [28]. When observations in one class are higher than the observation in other classes then there exists a class imbalance problem [29]. The unbalance incidence varies from 0.01 to 29.1%; that is, the percent of the data samples that belong to the positive class [30]. In the current analysis, for example, 9.5% of newborns had a low five-minute Apgar score. In this case, creating suitable testing and training data sets becomes difficult, since most classifiers are designed with the assumption that the test data is derived from the same distribution as the training data [31]. If the data set in imbalance, then one may get high accuracy just by predicting the majority class, but will fail to capture the minority class, which is most often the point of creating the model in the first place [32]. Simply put, with imbalanced data sets, an algorithm doesn’t get the necessary information about the minority class to make an accurate prediction. SMOTE, found in the “DMwR” package, and ROSE, located in the “rose” package, both found in R software, were utilized to balance categories in the training set in the current study. SMOTE divides the data set into active and inactive instances, from which the training and testing data sets are generated. The training data is partitioned into sub-samples with each sub-sample containing an equal number of instances from each class, except for last sub-sample [33]. The classification model is then fitted repeatedly on every sub-sample and the final result is a majority voting over all the sub-samples. ROSE is a package for binary imbalanced learning that uses smoothed bootstrapping to draw artificial samples from the feature space neighbourhood around the minority class [34]. In this package, we performed random oversampling (ROS), random undersampling (RUS) and eventually the hybrid of oversampling and undersampling techniques. A combination of over- and undersampling is a compromise between the two while producing ties for the minority examples when the original training set size is large and the imbalance is extreme [35].

### Machine learning analysis

We compared the performance of six common machine learning classifiers in predicting low Apgar score: random forest (RF), logistic regression (Lreg), Gaussian Naïve Bayes (NB), Artificial Neural Networks (ANN), Boosting, and Bagging. RF is a technique consisting of a large number of decision trees that operate as an ensemble. Each individual tree in the random forest spits out a class prediction and the class with the most votes become our model’s prediction [36]. Lreg is a machine learning algorithm used to predict the probability that an observation belongs to one of two possible classes [37]. We used the generalized linear model function found in “glm” package to execute logistic regression algorithm. Naive Bayes (NB) classifier applies Bayes’ theorem with the “naive” assumption of independence between every set of features, meaning that all features contribute independently to the probability of the target outcome [38]. We used “*naiveBayes*” function in R-package to fit NB models. Artificial Neural Network (ANN) is a computational model inspired by biological neural networks aiming to simulate the human brain. The algorithms learn from inputs, hidden and output layers which are interconnected to produce the desired outputs. The input units receive information based on the internal weighting system, and the neural network attempts to learn about them and eventually produce the desired results [39]. We used “nnet” package in R to implement ANN algorithm. Boosting is an ensemble meta-algorithm that combines weak learners to form a firm rule for classification by performing several iterations, which improves the prediction accuracy. These algorithms seek to improve the prediction power by training a sequence of weak models, each compensating for its predecessors’ weaknesses [40]. Bagging or Bootstrap aggregation also uses ensemble learning to evolve machine learning models. This algorithm is used with decision trees, where it significantly raises the stability of models by reducing variance and eliminating the challenge of model overfitting. Briefly, the base algorithm reads the data and assigns equal weight to each covariate under observation [41]. We evaluated all these models using the 30% hold-out method along with 10-fold cross validation to avoid potential model overfitting. To evaluate the models’ validity and performance, we used “area under the receiver operating characteristic curve” (AUC-ROC), precision, recall, F1 score, Matthews Correlation Coefficient (MCC), bookmaker informedness (BM), balanced accuracy (BA), and markedness (MK) as described in eqs. 1 through 8.

**Table:** 

Accuracy	$\frac{TP+ TN}{TP+ TN+ FP+ FN}$	(1)
Precision	$\frac{TP}{TP+ FP}$	(2)
Recall	$\frac{TP}{TP+ FN}$	(3)
F1 score	$\frac{2\ast Precision\ast Recall}{Precision+ Recall}$	(4)
MCC	$\frac{TP\ast TN- FP\ast FN}{\sqrt{\left( TP+ FP\right)\ast \left( TP+ FN\right)\ast \left( TN+ FP\right)\ast \left( TN+ FN\right)}}$	(5)
BA	$\frac{TP}{2\left( TP+ FN\right)}+\frac{TN}{2\left( TN+ FP\right)}$	(6)
BM	$\frac{TP}{TP+ FN}+\frac{TN}{TN+ FP}-1$	(7)
MK	$\frac{TP}{TP+ FP}+\frac{TN}{TN+ FN}-1$	(8)

Where: TP = True positives, TN = True negative, FP = False positive, FN = False negative, MCC = Matthews Correlation Coefficient, BA = Bookmaker informedness, MK = markedness.

### Decision curve analysis (DCA)

Additionally, model outputs were compared using a decision curve analysis with a set of threshold probabilities. DCA is a common framework in which a clinical judgment of the relative value of benefits and harms associated with the prediction model is made. It calculates the “net-benefit” as a parameter of interest for each threshold probability [42]. Simply put, the DCA incorporates the information about the benefits of correctly identifying the low Apgar scores (true positives) and the relative harm of incorrectly identifying the same (false positives). A model is said to be superior to another at the chosen threshold if its net benefit surpasses the net benefit of other models for a given value of threshold probability [43]. We, therefore, presented the net benefit of each model using the “dca” package in R tool. The net benefit for Net benefit is calculated as a weighted combination of true and false positives (see the formula hereunder) as shown in eq. 9.

9 $$\mathrm{Net}\ \mathrm{Benefit}=\frac{TP- FP\ast Pt\left/ 1- Pt\right.}{N}$$

Where: TP = True positives, FP = False positive, Pt = threshold probability, N = total number of observations.

## Results

The mean maternal age of study participants was 27 (SD = 6) years. More than half (61%) of deliveries were from mothers aged between 20 and 30 years. Sociodemographic and clinical characteristics of study participants are clearly displayed in Table 2.

**Table 2:** Demographic information of the study participant (*N* = 7716)

Maternal characteristics	Low (< 7) Apgar score	Normal (≥7) Apgar score	χ^**2**^ ***p***-value
**Parity**			
Nulliparous	409 (55.8)	3817 (54.66)	
Multiparous	324 (44.2)	3166 (45.34)	0.556
**Maternal age**			
< 25	273 (37.24)	2575 (36.88)	
25–35	361 (49.25)	3606 (51.64)	
> 35	99 (13.51)	802 (11.49)	0.214
**Gestational age**			
Term	463 (63.17)	5683 (81.38)	
Preterm	209 (28.51)	593 (8.49)	
Post term	61 (8.32)	707 (10.12)	< 0.001
**PROM**			
No	709 (96.73)	6829 (97.79)	
Yes	24 (3.27)	154 (2.21)	0.067
**Gestational diabetes**			
No	730 (99.59)	6974 (99.87)	
Yes	3 (0.41)	9 (0.13)	0.067
**Prenatal visits**			
< 3	296 (40.38)	1796 (25.72)	
3–6	365 (49.80)	3997 (57.24)	
> 6	72 (9.82)	1190 (17.04)	< 0.001
**Induction method**			
Oxytocin	591 (80.63)	6361 (91.09)	
Prostaglandins	142 (19.37)	622 (8.91)	< 0.001
**Referred for delivery**			
No	453 (61.80)	5573 (79.81)	
Yes	280 (38.20)	1410 (20.19)	< 0.001
**Ever Use of Family planning**			
No	344 (46.93)	2896 (41.47)	
Yes	389 (53.07)	4087 (58.53)	0.004
**Smoking during pregnancy**			
No	729 (99.45)	6966 (99.76)	
Yes	4 (0.55)	17 (0.24)	0.135
**Alcohol during pregnancy**			
No	550 (75.03)	4977 (71.27)	
Yes	183 (24.97)	2006 (28.73)	0.032
**Child sex**			
Female	412 (56.21)	3563 (51.02)	
Male	321 (43.79)	3420 (48.98)	0.008
**Body mass index**			
Underweight	2 (0.27)	27 (0.39)	
Normal	109 (14.87)	1262 (18.07)	
Overweight	455 (62.07)	4133 (59.19)	
Obese	167 (22.78)	1561 (22.35)	0.169
**Epilepsy**			
No	732 (99.86)	6961 (99.68)	
Yes	1 (0.14)	22 (0.32)	0.399
**Preeclampsia**			
No	717 (97.82)	6873 (98.42)	
Yes	16 (2.18)	110 (1.58)	0.217

### Feature importance

**Fig. 2:**
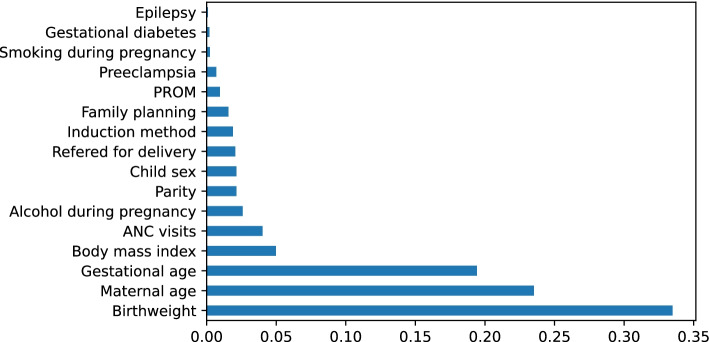
Feature importance measures as revealed by “Extra-tree classifier” for prediction of low Apgar scores following a successful labor induction intervention

We used “*Extra-tree classifier”* in Python to obtain significant attributes that play a significant role in predicting low Apgar score and we found that birthweight, maternal age, and gestational age to be the essential features (Fig. 2).

### Correlation matrix for predictors of low Apgar score following as successful labor induction (IOL)

**Fig. 3:**
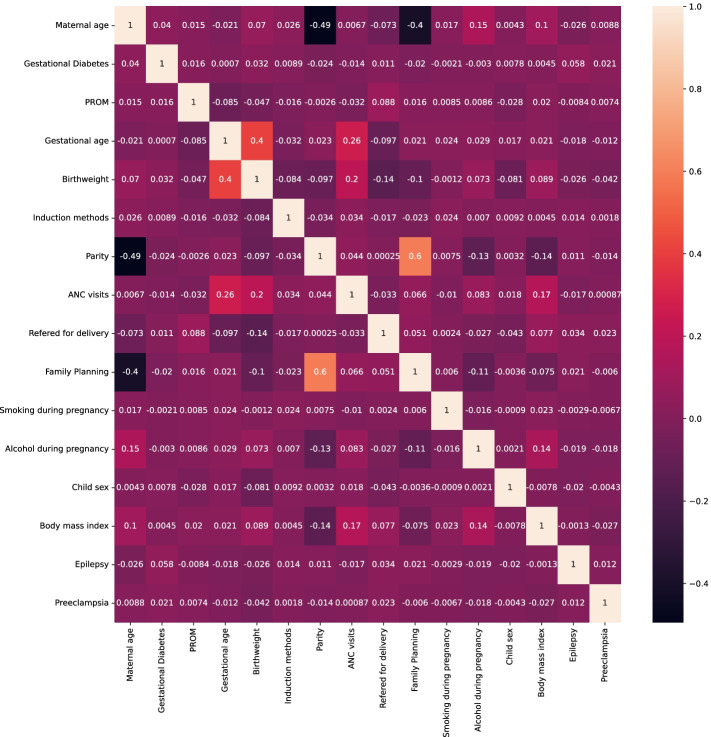
Heatmap showing correlation among predictors of low (< 7) Apgar score following IOL intervention

To assess the presence of correlated variables prior to model building, we quantified and visualized the corretion matrix using “*seaborn”* library in Python (Fig. 3). This plot presents the level of dependence among predictor variables and indicates a predictive relationship. Presence of correlated variables in a model may produce erroneous associations, leading to unreliable conclusions. Our data revealed only mild correlation (60%) between “Parity status” and “ever use of family planning” .

**Fig. 4:**
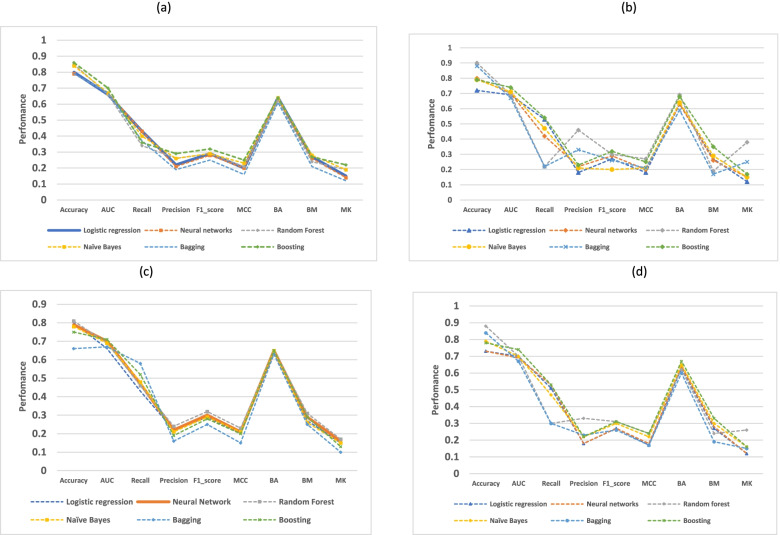
Graphical representation of performance metrics of the selected models following the application of (**a**) SMOTE (**b**) ROSE (oversampling) (**c**) ROSE (undersampling) (**d**) ROSE (Hybrid of oversampling and Oversampling

Table 3 and Fig. 4 summarizes the predictive performance of the chosen machine learning models. After resampling, recall, F1 ranking, BA, and BM all improved. However, the increase in recall was notable in comparison to the improvements in other metrics.

**Table 3:** Performance metrics for low Apgar score before and after applying SMOTE and ROSE resampling techniques

Algorithm	Metrics	Before resampling	SMOTE	ROSE (Oversampling)	ROSE (undersampling)	ROSE (Hybrid)
Logistic regression	Accuracy	0.91	0.80	0.72	0.80	0.73
	AUC	0.69	0.66	0.69	0.66	0.70
	Recall	0.12	0.43	0.53	0.43	0.51
	Precision	0.79	0.22	0.18	0.22	0.18
	F1-score	0.21	0.29	0.27	0.29	0.27
	MCC	0.29	0.20	0.18	0.20	0.17
	BA	0.56	0.64	0.63	0.64	0.63
	BM	0.11	0.27	0.27	0.27	0.27
	MK	0.71	0.15	0.12	0.15	0.12
Neural networks	Accuracy	0.92	0.79	0.80	0.79	0.73
	AUC	0.70	0.67	0.70	0.70	0.69
	Recall	0.16	0.43	0.42	0.47	0.53
	Precision	0.77	0.21	0.22	0.22	0.18
	F1-score	0.26	0.28	0.29	0.30	0.27
	MCC	0.33	0.20	0.20	0.21	0.18
	BA	0.58	0.63	0.63	0.65	0.64
	BM	0.15	0.26	0.26	0.29	0.28
	MK	0.70	0.14	0.15	0.16	0.12
Random forest	Accuracy	0.91	0.85	0.90	0.81	0.88
	AUC	0.68	0.66	0.69	0.69	0.70
	Recall	0.12	0.34	0.22	0.46	0.30
	Precision	0.84	0.26	0.46	0.24	0.33
	F1-score	0.21	0.29	0.30	0.32	0.31
	MCC	0.30	0.21	0.27	0.23	0.24
	BA	0.56	0.62	0.69	0.65	0.62
	BM	0.11	0.24	0.19	0.31	0.24
	MK	0.76	0.19	0.38	0.17	0.26
Naïve Bayes	Accuracy	0.91	0.84	0.79	0.78	0.79
	AUC	0.69	0.67	0.71	0.69	0.70
	Recall	0.25	0.40	0.47	0.48	0.47
	Precision	0.56	0.26	0.21	0.21	0.22
	F1-score	0.35	0.29	0.20	0.29	0.30
	MCC	0.33	0.23	0.21	0.21	0.22
	BA	0.61	0.64	0.64	0.65	0.65
	BM	0.23	0.28	0.29	0.26	0.30
	MK	0.49	0.19	0.15	0.15	0.16
Boosting	Accuracy	0.92	0.86	0.79	0.75	0.78
	AUC	0.73	0.70	0.74	0.71	0.74
	Recall	0.17	0.36	0.54	0.52	0.53
	Precision	0.78	0.29	0.23	0.19	0.22
	F1-score	0.28	0.32	0.32	0.28	0.31
	MCC	0.34	0.25	0.25	0.20	0.24
	BA	0.58	0.64	0.68	0.65	0.67
	BM	0.16	0.27	0.35	0.29	0.33
	MK	0.70	0.22	0.17	0.13	0.16
Bagging	Accuracy	0.91	0.79	0.88	0.66	0.84
	AUC	0.68	0.67	0.67	0.67	0.67
	Recall	0.19	0.37	0.22	0.58	0.30
	Precision	0.52	0.19	0.33	0.16	0.23
	F1-score	0.28	0.25	0.26	0.25	0.26
	MCC	0.27	0.16	0.21	0.15	0.17
	BA	0.58	0.61	0.59	0.63	0.60
	BM	0.17	0.21	0.17	0.25	0.19
	MK	0.44	0.12	0.25	0.10	0.15

**Fig. 5:**
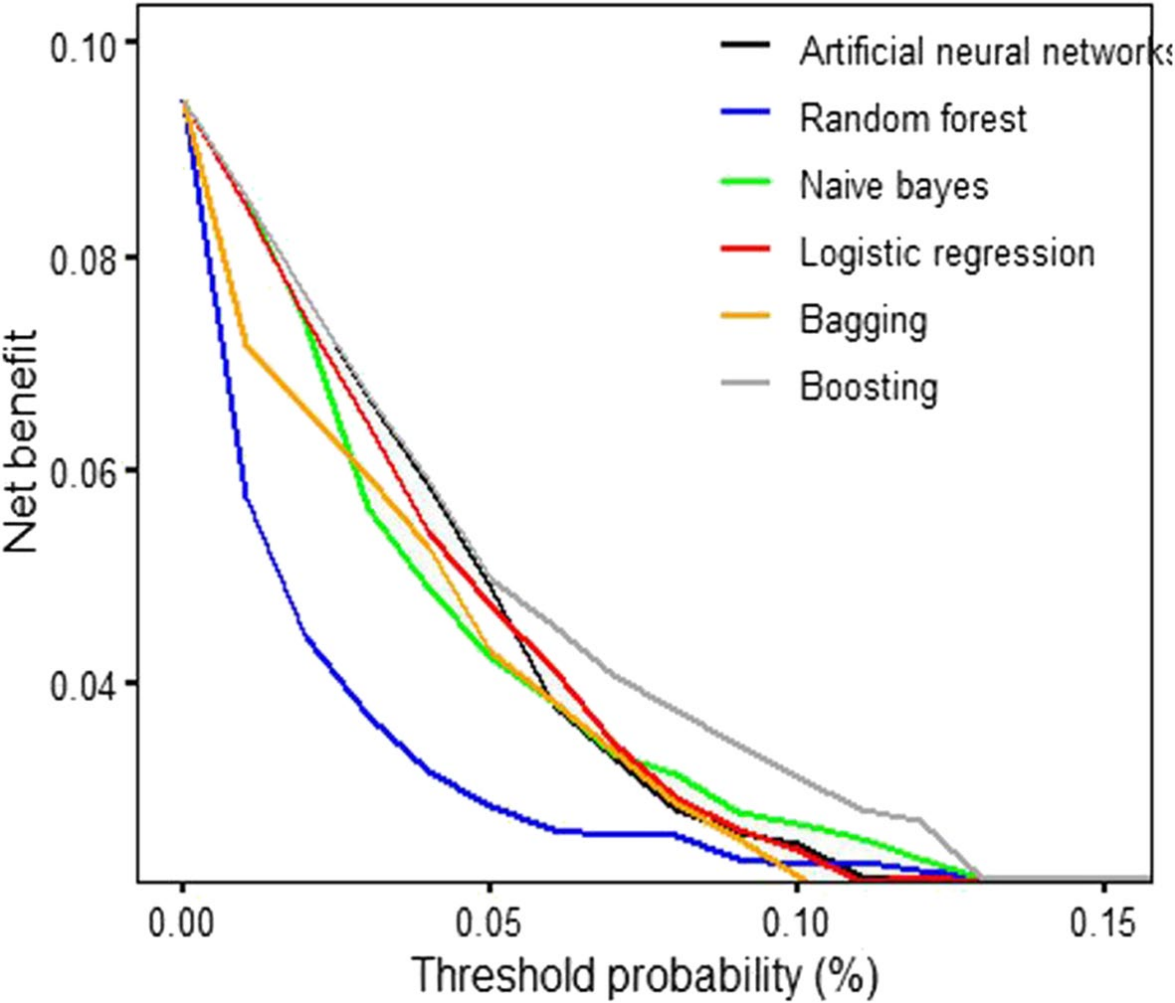
Decision curve analysis (DCA) for predictive models over the range of threshold probability

The DCA results (Fig. 5) show that the boosting algorithm outperformed all other models in terms of net benefit across the range of threshold probabilities.

### Model calibration and ROC curves for the classifiers

**Fig. 6:**
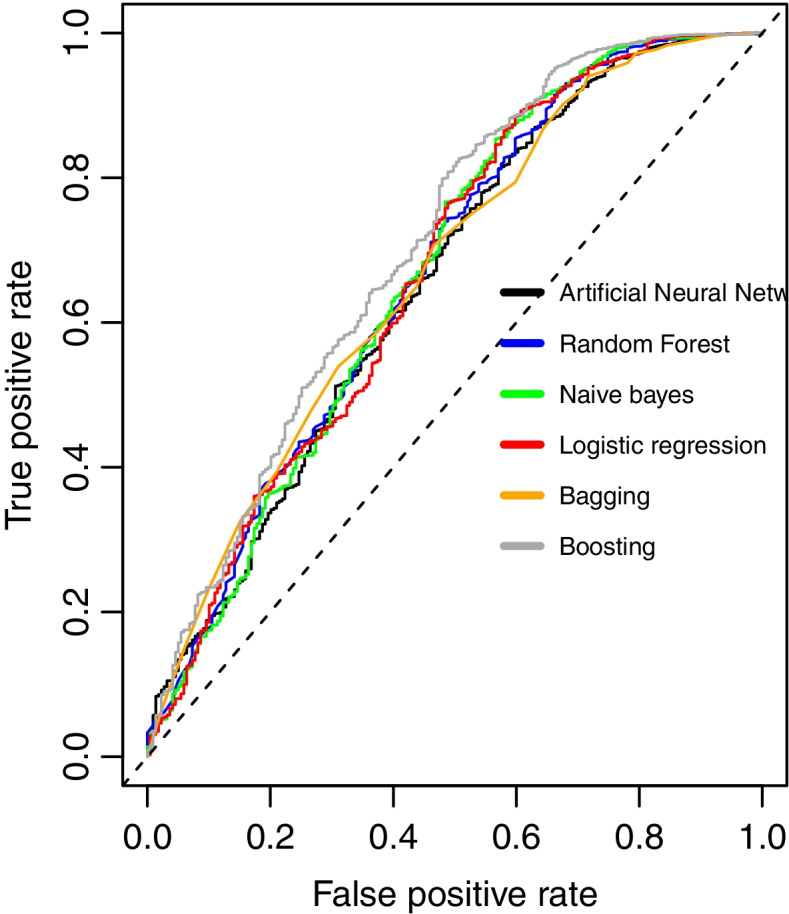
Receiver Operating Characteristic (ROC) curve for the baseline performance of the selected classifiers

Calibration curves or reliability diagrams were also applied to compare how well the probabilistic predictions of the selected binary classifier are calibrated. The x axis depicts the predicted probability on an average basis for each bin while the y axis represents the fraction of positives, or the proportion of samples classified as positive. We used “calibration_curve” package from “sklearn.calibration” module in Python to calculate the per bin average predicted probabilities and fraction of positives. Our outputs indicates that LogisticRegression generates probability predictions that are closer to optimal compared to any other algorithm tested. We also plotted the ROC curve to illustrate the diagnostic ability of a binary classifier system as its discrimination threshold is varied. In this occasion, boosting algorithm outperformed all other models before and after execution of resampling methods. ROC curve for the baseline performance of the classifiers are shown in Fig. 6 while the Python codes (with calibration outputs), R-syntax and TRIPOD (Transparent reporting of a multivariable prediction model for individual prognosis or diagnosis) statement are supplied as Supplementary materials.

## Discussion

Among the covariates in this database, birthweight, gestational age, and maternal age were identified as highly predictive of low Apgar score among induced vaginal deliveries. Studies have shown that newborns who weigh less than 2500 g have a greater chance to have low Apgar score than those born with appropriate weight [44]. Previous researchers also reported that respiratory efforts, muscle tone, and reflex were the major determinants for a decreasing Apgar score with declining gestational age [45]. The role of maternal age on neonatal outcomes has been reported in many studies. The rates of preterm delivery, NICU transfer, GDM (Gestational Diabetes Mellitus), placenta previa, induction failure, and primary cesarean section were progressively increased with increasing maternal age groups [46]. As neonates with low Apgar score were extremely low (9.5%) compared to those with normal scores (90.5%) in the current study, we studied the impact of class rebalancing methods on the performance of the selected ML classifiers in predicting low five-minute Apgar score. Prior research has used class rebalancing methods to improve the models’ performance [35, 47]. Studies in machine learning field have shown a performance increase when class rebalancing techniques are used. Studies have shown that using class rebalancing techniques resulted into an increase of AUC by up to 40%. For instance, Kamei et al. [48] demonstrated the performance improvement of class rebalancing techniques on two proprietary system defect datasets. However, the current study could not appreciate the improvement in terms of AUC following the application of ROSE and SMOTE rebalancing techniques. Our findings are consistent with those of Blagus et al. [49], who found that SMOTE does not perform well with high-dimensionality data. Riquelme et al. [50] contended that class rebalancing techniques have little impact on the overall efficiency of models trained on four NASA datasets. The current study has displayed and visualized the performance of other metrics including accuracy, precision, recall, F1 score, MCC, BA, BM and MK. However, there is an ongoing debate on which metrics are the most reliable and informative when it comes to reporting models’ performance on an imbalanced dataset [51]. We have seen that accuracy and BA score were maintained compared to all other metrics before and after applying the rebalancing techniques in all the models. Notable is the significant increase in recall scores across all models. The recall score, which is the ratio of correctly predicted positive observations to all observations in the actual class, indicates that the rebalanced classifiers could correctly predict more neonates with a low Apgar score, than the one before using SMOTE or ROSE methods. While most researchers believe that accuracy is the most appropriate performance metric [52], some studies have indicated that when the dataset is unbalanced, accuracy may not be a reliable measure anymore as it provides an overoptimistic estimation of the classifier ability on the majority class [53, 54]. Regarding the MCC and F1 score, Dubey and Tatar state that these two measures “provide more realistic estimates of real-world model performance” [55]. In addition, a study conducted by Guilford [56] has shown MCC to be an effective way especially in an imbalanced design. However, we observed a loss of MCC scores after employing resampling techniques in all of the chosen ML models. We can state here that lack of or little improvement on MCC F1 scores, precision, BA, BM and MK metrics means a decline values for all the four basic rates of the confusion matrix: true positive rate (TPR), true negative rate (TNR), positive predictive value (PPV), and negative predictive value (NPV). One of the potential reasons may be the so-called concept drift. The concept drift in predictive analytics and machine learning refers to how the statistical properties of the target variable, which the model is attempting to predict, change over time in unforeseen ways. This means there may be a change of in the relationships between the predictor variables and the outcome of interest over time, as a result, class rebalancing techniques can have an impact on learning process and hence leading to poor predictive performance on some of the metrics [57]. In the future studies, distinct methods for detecting concept drift may be required. Furthermore, studies have reported that overgeneralization, as it blindly generalizes the minority area without regard to the majority class, as well as lack of flexibility are potential challenges around SMOTE technique while ROSE has been shown to be prone to overfitting. Literature also suggests that oversampling using ROSE involves making exact copies of existing examples, a scenario which makes overfitting likely [58]. While generating synthetic examples, SMOTE does not take into consideration that the neighboring examples may originate from the other class, a scenario which increases the possibility of class overlapping that introduces additional noise and hence the likelihood of declined predictive performance [59]. We hence hope to extend the analysis into further exploration of techniques such as TOMEK links so as to handle overlapping that may have been introduced by resampling methods [60]. Other specialized resampling methods including cost-sensitive algorithms and ensemble methods will be deployed in our upcoming study [61]. The model calibration curve indicates that logistic regression has the best probability predictions that other models. This could be explained by the fact that logistic regression produces quite accurate probability predictions because it optimizes log-odds, which is simply a convenient restatement of class probability. In other words, probability is directly related to the cost function and thus the algorithm produces unbiased probability estimates [62]. This means Lreg model had returned a well calibrated predictions as it directly optimizes the “Log loss” (also known as cross-entropy loss) [63, 64]. In other words, the tested models returned somewhat a biased probabilities compared to that shown by logistic regression. Methods like as bagging and random forests that average predictions from a base set of models may have trouble making predictions near 0 and 1, as variance in the underlying base models may bias predictions that should be near 0 or 1 away from these values. Due to the fact that predictions are limited to the interval [0,1], variance-induced errors tend to be one-sided at zero and one. We furtherly used decision curve analysis (DCA) to portray the impact of false-negative and false-positive misclassification errors. If a model or test has the highest net benefit across the entire range of reasonable threshold probabilities, then clearly, that model should be considered for making a decision about the outcome. We observed that the net benefit for Boosting algorithm surpassed that of all other models, which mean higher recalls in predicting the likelihood of low Apgar score. In other words, DCA measures the impact of false-negative and false-positive misclassification errors. As supported by previous study [65] Boosting models surpassed all other models under investigation in terms of net benefit over the extended threshold probabilities. The net benefit metric provides information about the consequences of using the model in question. Taking the case where falsely predicting a case as “low apgar score” (false-negative) is much more harmful than a false-positive result, a model that has a much greater specificity but slightly lower sensitivity than another may have a higher performance metric, say AUC, but would be a poorer choice for clinical use. Simply put, applying random forest algorithm for predicting low Agar score in neonates using this registry database may be more clinically consequential than using any other ML algorithm tested in the current study.

### Strength and limitation

This article is the first empirical research to examine the impact of rebalancing methods on prediction of low-Apgar score after IOL intervention using widely used machine learning algorithms. We enrolled deliveries over a 15-year period, which may have included a diverse group of study participants with contrasting characteristics. Furthermore, neonatologist can consider the models and the different risk factors that are identified as important factors by these models in their decision making. Artificial intelligence researchers and developers who are interested in developing predictive models or decision support systems for neonatal outcomes can also use the results of this study to select the best models for the prediction of low Apgar score. However, our study had some limitations that should be taken into consideration during interpretations of the results. Changes in protocols over time may have influenced the mode of delivery and variability of Apgar scores. All observations with missing values in both the outcome and predictors were excluded from the analyses. We claim that this may not be the best way to handle missing data since critical information can be lost when incomplete rows of data are discarded. However, learning algorithms are significantly affected by missing values as they rely heavily on data to learn the underlying input-output relationships of the attributes being modeled. Including subjects with missing values would bias the performance metrics under observation in this instance. Further studies that will consider techniques for handling missing data prior to assessing predictive performance of ML methods are warranted so as to avoid potential information leakage.

## Conclusion

Maternal and neonatal healthcare department should recognize the role played by birthweight, maternal age, and gestational age in predicting low Apgar score in vaginal delivery following labor induction. The study recommends the use of Boosting algorithms in predicting low Apgar score as it showed the best performance as well as extended net benefits despite the imbalanced nature of the dataset. Our findings suggest that the effect of class rebalancing techniques on the performance of prediction models may be context dependent since the rebalancing techniques substantially improved “Recall scores” while showing no significant impact on other metrics. With regard to unbalanced data, we believe that future study should concentrate on revealing the role played by data structure on the performance of learning algorithms while stressing on exploring the robust algorithms that can learn from a wide spectrum of data structure presented. Furthermore, we think it is important to explore the relationship between data imbalanced ratio and the complexity of the learning model, that is, identifying the best levels of balanced ratio for a given learning algorithm. We believe that thorough understanding of these queries will not only provide fundamental insights into imbalanced learning problem, but also provide critical technical tools and solutions to many practical real imbalanced learning applications.

## Supplementary Information

**Additional file 1.**

## Data Availability

The dataset analyzed during the current study are not publicly available in order to protect the participants’ anonymity but is available from the corresponding author on reasonable request.

## Abbreviations

ACC: Accuracy
AUC: Area under curve
ANN: Artificial neural network
BA: Balanced Accuracy
BM: Bookmaker Informedness
DCA: Decision curve analysis
IOL: Induction of labor
KCMC: Kilimanjaro Christian Medical Center
MK: Markedness
ML: Machine learning
NB: Naïve Bayes
NPV: Negative predictive value
PPV: Positive predictive value
RF: Random forest
ROSE: Random Oversampling Examples
SMOTE: Synthetic Minority Oversampling Technique
