# Supplementary material for: Prediction of low Apgar score at five minutes following labor induction intervention in vaginal deliveries: machine learning approach for imbalanced data at a tertiary hospital in North Tanzania
Source: BMC Pregnancy Childbirth. 2022 Apr 1;22:275. doi: 10.1186/s12884-022-04534-0 (PMC8976377; doi:10.1186/s12884-022-04534-0)

In [1]:
```python
import pandas as pd
import numpy as np
from sklearn.model_selection import train_test_split
from sklearn.ensemble import AdaBoostClassifier
from sklearn.metrics import classification_report
from sklearn.metrics import average_precision_score
from sklearn.metrics import precision_recall_curve
from sklearn.utils import resample
from imblearn.over_sampling import SMOTE
from sklearn.metrics import confusion_matrix
from sklearn.metrics import roc_curve, auc
import matplotlib.pyplot as plt
from imblearn import under_sampling
```

In [2]:
```python
 #read in data and set target variable
df = pd.read_csv("C:/Users/silve/Desktop/Project_3/attempt_1.csv")
df.head()
```

Out[2]:

	Maternal_age	Gestational_diabetes	PROM	Gestational_age	Birthweight	Induction_method	Parity	ANC_visits	Refered_for_delivery	Family_planning	Smoking_during_pregnancy	Alcol
**0**	37	0	0	40	5.90	1	0	1	0	0	1	
**1**	29	0	1	40	2.50	1	1	2	1	1	0	
**2**	30	0	1	40	3.25	1	1	2	0	1	0	
**3**	26	0	0	40	3.10	1	0	2	0	0	0	
**4**	28	0	0	38	2.40	1	0	2	0	0	0	

In [3]:
```python
df.shape
```

Out[3]: (7716, 17)

In [4]:
```python
X=df.iloc[:,0:16]
y=df.iloc[:,16]
```

In [5]:
```python
X_train, X_test, y_train, y_test = train_test_split(X, y,
                                                    test_size=0.3,
                                                    random_state=42)
```

In [6]:
```python
from collections import Counter

counter = Counter(df['Apgar_score'])
print(counter)
```

```
Counter({0: 6983, 1: 733})
```

In [7]:
```python
##RANDOM FOREST CLASSIFIER#####
from sklearn import ensemble
rf=ensemble.RandomForestClassifier(n_estimators=100)
rf.fit(X_train, y_train) #Train
rf.score(X_test, y_test) #Make predictions
y_pred = rf.predict(X_test)
rf.score(X_test, y_test) #Make predictions
confusion_matrix(y_test, y_pred)
```

Out[7]:
```
array([[2075,   15],
       [ 182,   43]], dtype=int64)
```

In [8]:
```python
##NAIVE BAYES CLASSIFIER####
from sklearn.naive_bayes import GaussianNB
nb =  GaussianNB()
nb.fit(X_train, y_train)
nb.score(X_test, y_test) #Make predictions
y_pred = nb.predict(X_test)
nb.score(X_test, y_test) #Make predictions
confusion_matrix(y_test, y_pred)
```

Out[8]:
```
array([[1985,  105],
       [ 165,   60]], dtype=int64)
```

In [9]:
```python
##LOGISTIC REGRESSION####
from sklearn.linear_model import LogisticRegression
lr=LogisticRegression()
lr.fit(X_train, y_train)
lr.score(X_test, y_test) #Make predictions
y_pred = lr.predict(X_test)
lr.score(X_test, y_test) #Make predictions
confusion_matrix(y_test, y_pred)
```

```
c:\users\silve\appdata\local\programs\python\python38\lib\site-packages\sklearn\linear_model\_logistic.py:762: ConvergenceWarning: lbfgs failed to
converge (status=1):
STOP: TOTAL NO. of ITERATIONS REACHED LIMIT.

Increase the number of iterations (max_iter) or scale the data as shown in:
    https://scikit-learn.org/stable/modules/preprocessing.html
Please also refer to the documentation for alternative solver options:
    https://scikit-learn.org/stable/modules/linear_model.html#logistic-regression
  n_iter_i = _check_optimize_result(
```

Out[9]:
```
array([[2083,    7],
       [ 191,   34]], dtype=int64)
```

In [10]:
```python
#BAGGING CLASSIFIER###
from sklearn.ensemble import BaggingClassifier
bg = BaggingClassifier()
bg.fit(X_train, y_train)
bg.score(X_test, y_test) #Make predictions
y_pred = bg.predict(X_test)
bg.score(X_test, y_test) #Make predictions
confusion_matrix(y_test, y_pred)
```

Out[10]:
```
array([[2045,   45],
       [ 180,   45]], dtype=int64)
```

In [11]:
```python
##BOOSTING CLASSIFIER####
from sklearn.ensemble import GradientBoostingClassifier
boost = BaggingClassifier()
boost.fit(X_train, y_train)
boost.score(X_test, y_test) #Make predictions
y_pred = boost.predict(X_test)
boost.score(X_test, y_test) #Make predictions
confusion_matrix(y_test, y_pred)
```

Out[11]:
```
array([[2062,   28],
       [ 179,   46]], dtype=int64)
```

In [12]:
```python
##Probs
r_probs = [0 for _ in range (len(y_test))]
rf_probs = rf.predict_proba(X_test)[:, 1]
nb_probs = nb.predict_proba(X_test)[:, 1]
lr_probs = lr.predict_proba(X_test)[:, 1]
bg_probs = bg.predict_proba(X_test)[:, 1]
boost_probs = boost.predict_proba(X_test)[:, 1]
```

In [13]:
```python
from sklearn.calibration import CalibratedClassifierCV
import matplotlib.pyplot as plt
```

In [14]:
```python
from sklearn.datasets import make_classification
from sklearn.calibration import calibration_curve
#####################Creating Calibration Curve for RANDOM FOREST###########################################

x, y = calibration_curve(y_test, rf_probs, n_bins = 10, normalize = True)
# Plot calibration curve

# Plot perfectly calibrated
plt.plot([0, 1], [0, 1], linestyle = '--', label = 'Ideally Calibrated')

# Plot model's calibration curve
plt.plot(y, x, marker = '.', label = 'Random Forest Classifier')

leg = plt.legend(loc = 'upper left')
plt.xlabel('Average Predicted Probability in each bin')
plt.ylabel('Ratio of positives')
plt.show()
```

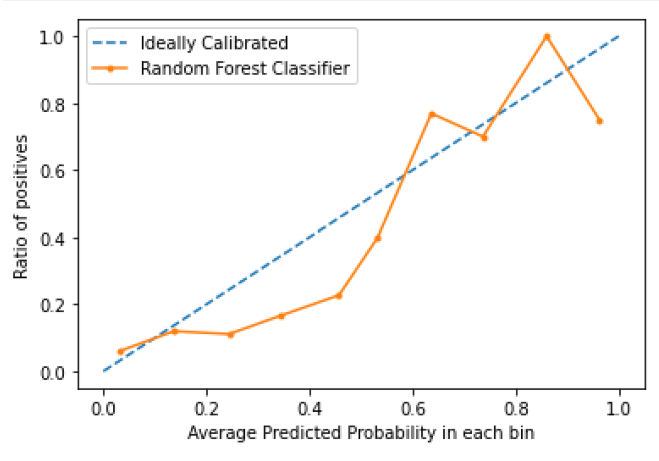

In [15]:
```python
#####################Creating Calibration Curve for RANDOM FOREST###########################################

x, y = calibration_curve(y_test, lr_probs, n_bins = 10, normalize = True)
# Plot calibration curve

# Plot perfectly calibrated
plt.plot([0, 1], [0, 1], linestyle = '--', label = 'Ideally Calibrated')

# Plot model's calibration curve
plt.plot(y, x, marker = '.', label = 'Logistic Regression Classifier')

leg = plt.legend(loc = 'upper left')
plt.xlabel('Average Predicted Probability in each bin')
plt.ylabel('Ratio of positives')
plt.show()
```

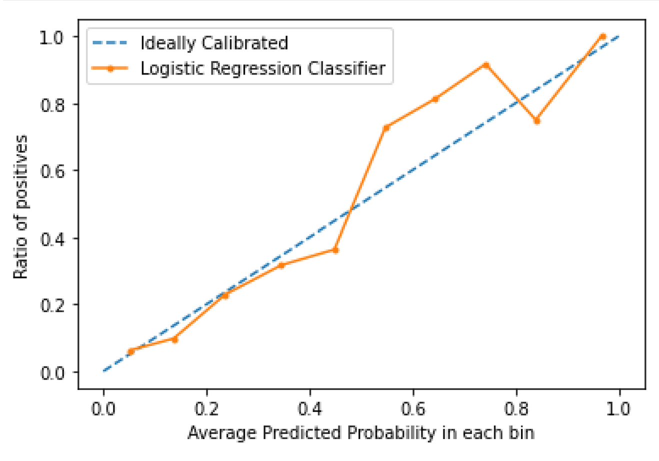

In [16]:
```python
#####################Creating Calibration Curve for NAIVE BAYES CLASSIFIER###########################################

x, y = calibration_curve(y_test, nb_probs, n_bins = 10, normalize = True)
# Plot calibration curve

# Plot perfectly calibrated
plt.plot([0, 1], [0, 1], linestyle = '--', label = 'Ideally Calibrated')

# Plot model's calibration curve
plt.plot(y, x, marker = '.', label = 'Naive Bayes Classifier')

leg = plt.legend(loc = 'upper left')
plt.xlabel('Average Predicted Probability in each bin')
plt.ylabel('Ratio of positives')
plt.show()
```

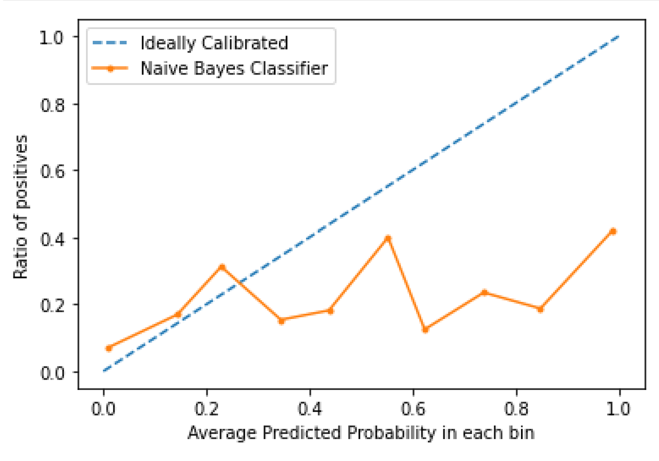

In [17]:
```python
#####################Creating Calibration Curve for BAGGING###########################################

x, y = calibration_curve(y_test, bg_probs, n_bins = 10, normalize = True)
# Plot calibration curve

# Plot perfectly calibrated
plt.plot([0, 1], [0, 1], linestyle = '--', label = 'Ideally Calibrated')

# Plot model's calibration curve
plt.plot(y, x, marker = '.', label = 'Bagging Classifier')

leg = plt.legend(loc = 'upper left')
plt.xlabel('Average Predicted Probability in each bin')
plt.ylabel('Ratio of positives')
plt.show()
```

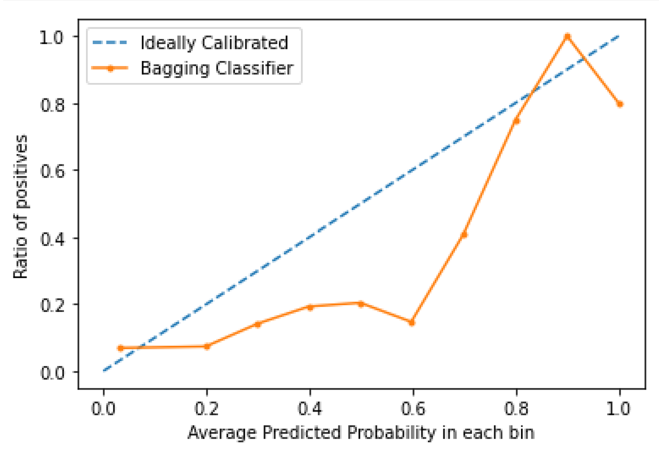

In [18]:
```python
#####################Creating Calibration Curve for BOOSTING CLASSIFIER###########################################

x, y = calibration_curve(y_test, boost_probs, n_bins = 10, normalize = True)
# Plot calibration curve

# Plot perfectly calibrated
plt.plot([0, 1], [0, 1], linestyle = '--', label = 'Ideally Calibrated')

# Plot model's calibration curve
plt.plot(y, x, marker = '.', label = 'Boosting Classifier')

leg = plt.legend(loc = 'upper left')
plt.xlabel('Average Predicted Probability in each bin')
plt.ylabel('Ratio of positives')
plt.show()
```

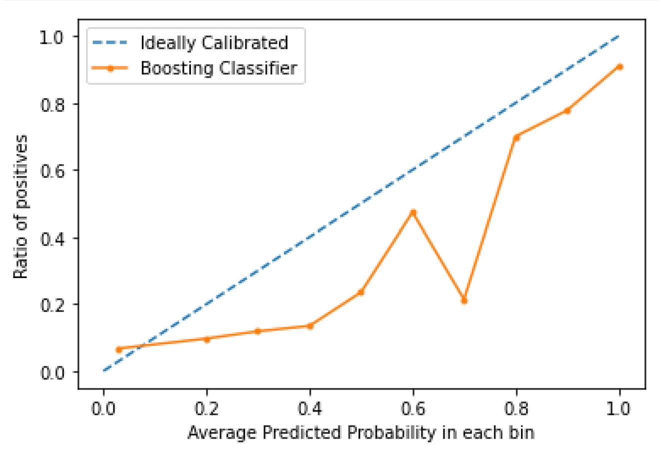

Supplement: Supplementary file 1 — Additional file 1. [file 12884_2022_4534_MOESM1_ESM.zip › Python_Calibration_curves.pdf]
