# Supplementary material for: Prediction of low Apgar score at five minutes following labor induction intervention in vaginal deliveries: machine learning approach for imbalanced data at a tertiary hospital in North Tanzania
Source: BMC Pregnancy Childbirth. 2022 Apr 1;22:275. doi: 10.1186/s12884-022-04534-0 (PMC8976377; doi:10.1186/s12884-022-04534-0)
Supplement: Supplementary file 1 — Additional file 1. [file 12884_2022_4534_MOESM1_ESM.zip › R_codes.pdf]

```
# input Stata file
library(foreign)
library(readstata13)
data<-read.dta13("C:/Users/silve/Desktop/Project_3/attempt_1.dta", nonint.factors = T)
utils::View(head(data))
summary(data)
View(data)

str(data)
#Divide data into training and testing sets
library(caret)
set.seed(100) # For reproducabilty
trainIndex <- createDataPartition(data$Apgar_score,p=.70,list=FALSE)
trainData <- data[trainIndex,]
testData <- data[-trainIndex,]
table(data$Apgar_score)
table(trainData$Apgar_score)
table(testData$Apgar_score)
#####################################################################################

#Boruta
#library(Boruta)
#boruta<-Boruta(Apgar_score~.,data=data, doTrace=2, maxRuns=500)
#print(boruta)
#plot(boruta, las=2, cex.axis=0.7)
#plotImpHistory(boruta)
#plot(boruta, las=2, cex.axis=0.6)


# 1) Logistic regression
```

```
library(DMwR)
ctrlLReg <- trainControl(## 10-fold CV
  method = "cv",
  number = 10,
  classProbs=TRUE,
  savePredictions = TRUE, sampling = "smote",
  allowParallel = TRUE, summaryFunction = twoClassSummary)

# Train
set.seed(1239)
lreg <- train(x=trainData[-which(names(trainData) %in% c("Apgar_score"))],
            y= trainData$Apgar_score, method="glm",family=binomial(),
            trControl=ctrlLReg)
lreg

# Test
set.seed(1239)
lreg.pred <- predict(lreg, testData[,-which(names(testData) %in% c("Apgar_score"))])
lreg.tab = table(pred = lreg.pred, true = testData[,c("Apgar_score")])
lreg.Conf = confusionMatrix(lreg.pred, testData[,c("Apgar_score")], positive =
                        levels(testData[,c("Apgar_score")])[2])
lreg.Conf



# 2) Naive Bayes
##################################################################################
# Load required packages
library(dplyr) # Used by caret
#library(nnet) # support vector machine
library(pROC) # plot the ROC curves
#library(e1071)
```

```
# install.packages("naivebayes")
library(naivebayes)

set.seed(1236)
# Setup for cross validation
ctrlNB <- trainControl(method="CV", # 10fold cross validation
                       number = 10,
                       savePredictions = TRUE,
                       classProbs=TRUE,
                       allowParallel = TRUE, sampling = "smote",
                       summaryFunction = twoClassSummary)

# Train
NBModel.tune <- train(x=trainData[-which(names(trainData) %in% c("Apgar_score"))],
                      y= trainData$Apgar_score,
                      method = "naive_bayes", # Naive Bayes
                      #tuneLength = 5, # 9 values of the cost function
                      #preProc = c("center","scale"), # Center and scale data
                      #not needed for this analysis
                      metric="ROC",
                      trControl=ctrlNB)
NBModel.tune


# Test
NBModel.pred <- predict(NBModel.tune, testData[,-which(names(testData) %in%
                                      c("Apgar_score"))])
NBModel.tab = table(pred = NBModel.pred, true = testData[,c("Apgar_score")])
NBModel.Conf = confusionMatrix(NBModel.pred, testData[,c("Apgar_score")], positive =
                     levels(testData[,c("Apgar_score")])[2])
NBModel.Conf
```

```
# 3) Artificial nueral network (ANN)
################################################################################
# Load important packages
library(nnet) # support vector machine
# library(pROC) # plot the ROC curves


set.seed(1234)
#set.seed(123)
# Setup for cross validation
ctrlANN <- trainControl(method="CV", # 10fold cross validation
                     number = 10,
                     #savePredictions = TRUE,
                     classProbs=TRUE,
                     allowParallel = TRUE,
                     savePredictions = TRUE, sampling="smote",
                     summaryFunction = twoClassSummary)
# Train: Artificial nueral network (ANN) model
set.seed(1234)
ANNModel.tune <- train(x=trainData[-which(names(trainData) %in% c("Apgar_score"))],
                  y= trainData$Apgar_score,
                  method = "nnet", # nueral network
                  #tuneLength = 5, # 9 values of the cost function
                  #preProc = c("center","scale"), # Center and scale data
                  #metric="ROC",
                  #linout=TRUE,
                  #maxit = 1000,
                  #tuneGrid = my.grid,
                  trace = T,
```

```
                #tuneLength = 10,
                #linout = 1,
                trControl=ctrlANN,
                metric="ROC")
ANNModel.tune
plot(ANNModel.tune)

# Test
set.seed(1234)
ANNModel.pred <- predict(ANNModel.tune, testData[,-which(names(testData) %in%
                                              c("Apgar_score"))])
ANNModel.tab = table(pred = ANNModel.pred, true = testData[,c("Apgar_score")])
ANNModel.Conf = confusionMatrix(ANNModel.pred, testData[,c("Apgar_score")],
                        positive = levels(testData[,c("Apgar_score")])[2])
ANNModel.Conf

# 4) Bagged Tree
################################################################################
# Specify the training configuration
ctrlBagTree <- trainControl(method = "cv",
                     number = 10,
                     classProbs=TRUE,
                     savePredictions = TRUE,
                     allowParallel = TRUE, sampling="smote",

                     summaryFunction = twoClassSummary) # For AUC
# Cross validate the credit model using "treebag" method;
# Track AUC (Area under the ROC curve)
set.seed(1237) # for reproducibility
BaggedTree <- train(x=trainData[-which(names(trainData) %in% c("Apgar_score"))],
                y= trainData$Apgar_score,
```

```
                method = "treebag",
                metric = "ROC",
                trControl = ctrlBagTree)
BaggedTree

# Test
set.seed(1237)
BaggedTree.pred <- predict(BaggedTree, testData[,-which(names(testData) %in%
                                          c("Apgar_score"))])
BaggedTree.tab = table(pred = BaggedTree.pred, true = testData[,c("Apgar_score")])
BaggedTree.Conf = confusionMatrix(BaggedTree.pred, testData[,c("Apgar_score")], positive =
                         levels(testData[,c("Apgar_score")])[2])
BaggedTree.Conf



# 5) Boosting
##########################################################################################
#install gbm package
# install.packages("gbm")
library(gbm)
# Specify the training configuration
ctrlBoosting <- trainControl(method = "cv",
                      number = 10,
                      classProbs=TRUE, sampling = "smote",
                      savePredictions = TRUE,allowParallel = TRUE,

                      summaryFunction = twoClassSummary) # For AUC

# Cross validate the credit model using "treebag" method;
# Track AUC (Area under the ROC curve)
set.seed(1238) # for reproducibility
```

```
Boosting <- train(x=trainData[-which(names(trainData) %in% c("Apgar_score"))],
              y= trainData$Apgar_score,
              method = "gbm",
              metric = "ROC",
              trControl = ctrlBoosting)
plot(Boosting)

# Test
set.seed(1238)
Boosting.pred <- predict(Boosting, testData[,-which(names(testData) %in% c("Apgar_score"))])
Boosting.tab = table(pred = Boosting.pred, true = testData[,c("Apgar_score")])
Boosting.Conf = confusionMatrix(Boosting.pred, testData[,c("Apgar_score")], positive =
                    levels(testData[,c("Apgar_score")])[2])
Boosting.Conf

#Load additional libraries
library(caTools)
library(mlbench)
#set.seed(123)
# Setup for cross validation
ctrlRF <- trainControl(method="CV", # 10fold cross validation
                  number = 10,
                  savePredictions = TRUE,
                  classProbs=TRUE,
                  allowParallel = TRUE, sampling = "smote",
                  summaryFunction = twoClassSummary)


#Train
set.seed(1235)
RFModel.tune <- train(x=trainData[-which(names(trainData) %in% c("Apgar_score"))],
```

```
                y= trainData$Apgar_score,
                method = "rf", # Random Forest
                #tuneLength = 5, # 9 values of the cost function
                #preProc = c("center","scale"), # Center and scale data
                metric="ROC",
                trControl=ctrlRF)
RFModel.tune
plot(RFModel.tune)
a<-varImp(RFModel.tune)
a
plot(a)
barplot(table(a))
# Test
set.seed(1235)
RFModel.pred <- predict(RFModel.tune, testData[,-which(names(testData) %in% c("Apgar_score"))])
RFModel.tab = table(pred = RFModel.pred, true = testData[,c("Apgar_score")])
RFModel.Conf = confusionMatrix(RFModel.pred, testData[,c("Apgar_score")], positive =
                      levels(testData[,c("Apgar_score")])[2])
RFModel.Conf



# RESULTS
###################################################################################
# Plot ROC curves
# =================================================================================
library(ROCR)
probANN <- predict(ANNModel.tune, newdata=testData[,-which(names(testData) %in%
                                    c("Apgar_score"))], type="prob")
predANN <- prediction(probANN[,1], testData[,c("Apgar_score")])
perfANN <- performance(predANN, measure = "tpr", x.measure = "fpr")
```

```
plot(perfANN, col="Black", lwd=1)

probRF <- predict(RFModel.tune, newdata=testData[,-which(names(testData) %in%
c("Apgar_score"))], type="prob")
predRF <- prediction(probRF[,1], testData[,c("Apgar_score")])
perfRF <- performance(predRF, measure = "tpr", x.measure = "fpr")
plot(perfRF, col="Blue", lwd=1, add = TRUE)

probNB <- predict(NBModel.tune, newdata=testData[,-which(names(testData) %in%
                                        c("Apgar_score"))], type="prob")
predNB <- prediction(probNB[,1], testData[,c("Apgar_score")])
perfNB <- performance(predNB, measure = "tpr", x.measure = "fpr")
plot(perfNB, col="Green", lwd=1, add = TRUE)

probLreg <- predict(lreg, newdata=testData[,-which(names(testData) %in% c("Apgar_score"))],
             type="prob")
predLreg <- prediction(probLreg[,1], testData[,c("Apgar_score")])
perfLreg <- performance(predLreg, measure = "tpr", x.measure = "fpr")
plot(perfLreg, col="Red", lwd=1, add = TRUE)

probBaggedTrees <- predict(BaggedTree, newdata=testData[,-which(names(testData) %in%
                                             c("Apgar_score"))], type="prob")
predBaggedTrees <- prediction(probBaggedTrees[,1], testData[,c("Apgar_score")])
perfBaggedTrees <- performance(predBaggedTrees, measure = "tpr", x.measure = "fpr")
```

```
plot(perfBaggedTrees, col="Orange", lwd=1, add = TRUE)



probBoosting <- predict(Boosting, newdata=testData[,-which(names(testData) %in%
                                    c("Apgar_score"))], type="prob")
predBoosting <- prediction(probBoosting[,1], testData[,c("Apgar_score")])
perfBoosting <- performance(predBoosting, measure = "tpr", x.measure = "fpr")
plot(perfBoosting, col="DarkGray", lwd=1, add = TRUE)



#all plots together
par(mar=c(5,5,1,1), cex=0.9) # cex = text font size, mar=margins (left, bottom, top, right)
plot(perfANN, col="Black", lwd=1)
plot(perfRF, col="Blue", lwd=1, add = TRUE)
plot(perfNB, col="Green", lwd=1, add = TRUE)
plot(perfLreg, col="Red", lwd=1, add = TRUE)
plot(perfBaggedTrees, col="Orange", lwd=1, add = TRUE)
plot(perfBoosting, col="DarkGray", lwd=1, add = TRUE)
abline(a=0, b=1, lwd=1, lty=2, col="Black")
legend(0.55,0.7, legend = c("Artificial neural networks", "Random forest", "Naive bayes",
                 "Logistic regression", "Bagging", "Boosting"),
    col = c("Black", "Blue", "Green", "Red", "Orange", "DarkGray"),
    lty = 1,lwd = 3,bty = "n", y.intersp = 1.5, cex=0.8)

#Compute AUC values
#==========================================================================
ANN_auc <- performance(predANN, measure = "auc")
ANN_auc <- ANN_auc@y.values[[1]]
ANN_auc
RF_auc <- performance(predRF, measure = "auc")
RF_auc <- RF_auc@y.values[[1]]
```

```
RF_auc
NB_auc <- performance(predNB , measure = "auc")
NB_auc <- NB_auc@y.values[[1]]
NB_auc
Lreg_auc <- performance(predLreg , measure = "auc")
Lreg_auc <- Lreg_auc@y.values[[1]]

Bagged_auc <- performance(predBaggedTrees , measure = "auc")
Bagged_auc <- Bagged_auc@y.values[[1]]
Bagged_auc

Boosting_auc <- performance(predBoosting , measure = "auc")
Boosting_auc <- Boosting_auc@y.values[[1]]
Boosting_auc


#an alternative and short way compared to the above syntax
aucANN<-auc(as.numeric(testData$Apgar_score), probANN[,2])#ANN
aucANN

aucRF<-auc(as.numeric(testData$Apgar_score), probRF[,2])#RF
aucRF

aucNB<-auc(as.numeric(testData$Apgar_score), probNB[,2])#NB
aucNB

aucLreg<-auc(as.numeric(testData$Apgar_score), probLreg[,2])#Lreg
aucLreg

aucBagging<-auc(as.numeric(testData$Apgar_score), probBaggedTrees[,2])#Bagging
aucBagging
```

```
aucBoosting<-auc(as.numeric(testData$Apgar_score), probBoosting[,1])#Boosting
aucBoosting


#Compute CI for AUC
#==============================================================================
library(pROC)
ci.auc(as.numeric(testData$Apgar_score), probANN[,2])#ANN
ci.auc(as.numeric(testData$Apgar_score), probRF[,2])#RF
ci.auc(as.numeric(testData$Apgar_score), probNB[,2])#NB
ci.auc(as.numeric(testData$Apgar_score), probLreg[,2])#Lreg
ci.auc(as.numeric(testData$Apgar_score), probBaggedTrees[,2])#Bagging
ci.auc(as.numeric(testData$Apgar_score), probBoosting[,2])#Boosting

#Comparison of AUC for different machines using Delong's Test
library(ROCR)
roc.test(aucLreg, aucANN, method = "delong") #delong test is the default
roc.test(aucLreg, aucNB)
roc.test(aucLreg, aucBagging)
roc.test(aucLreg, aucBoosting)
roc.test(aucLreg, aucRF)

#==============================================================================
#Compute CT for Sens, Spec, Prevelance ... etc
#==============================================================================
install.packages("epiR")
library(epiR)
epi.tests(ANNModel.Conf$table, conf.level = 0.95)
epi.tests(RFModel.Conf$table, conf.level = 0.95)
```

```
epi.tests(NBModel.Conf$table, conf.level = 0.95)
epi.tests(lreg.Conf$table, conf.level = 0.95)
epi.tests(BaggedTree.Conf$table, conf.level = 0.95)
epi.tests(Boosting.Conf$table, conf.level = 0.95)

# Decision curve analysis
# DCA analysis
# ==========================================================================
library("tidyverse")
library("dplyr")
#Predict Perinatal death
ANNModel.pred <- predict(ANNModel.tune, testData[,-which(names(testData) %in%
                                    c("Apgar_score"))], type = "prob")
RFModel.pred <- predict(RFModel.tune, testData[,-which(names(testData) %in% c("Apgar_score"))],
             type = "prob")
NBModel.pred <- predict(NBModel.tune, testData[,-which(names(testData) %in%
                                    c("Apgar_score"))], type = "prob")
lreg.pred <- predict(lreg, testData[,-which(names(testData) %in% c("Apgar_score"))], type = "prob")
BaggedTree.pred <- predict(BaggedTree, testData[,-which(names(testData) %in%
                                    c("Apgar_score"))], type = "prob")
Boosting.pred <- predict(Boosting, testData[,-which(names(testData) %in% c("Apgar_score"))], type
              = "prob")
dcadata <- testData %>%select(c(Apgar_score))
source("C:/Users/silve/Desktop/Project_3/dca.r")
# This file is avaible at http://www.decisioncurveanalysis.org
library(reshape2)
dcadata$ANN<-as.numeric(ANNModel.pred$Bad)

dcadata$RF<-as.numeric(RFModel.pred$Bad)
dcadata$NB<-as.numeric(NBModel.pred$Bad)
dcadata$Lreg<-as.numeric(lreg.pred$Bad)
```

```
dcadata$Bagging<-as.numeric(BaggedTree.pred$Bad)

dcadata$Boosting <- as.numeric(Boosting.pred$Bad)

data.set <- dcadata

attach(data.set)

data.set$Apgar_score<-as.numeric(data.set$Apgar_score)

data.set$Apgar_score<-data.set$Apgar_score-1 #0=spont, 1=Bad (in the testing data)


#Plot the DCA curves

library(DCA)

dca(data=data.set, outcome="Apgar_score",

   predictors=c("ANN", "RF", "NB", "Lreg", "Bagging", "Boosting"), xstart=0, ymin=0)

dcaoutput <- dca(data=data.set, outcome="Apgar_score",

               predictors=c("ANN", "RF", "NB", "Lreg", "Bagging", "Boosting"), xstart=0,xstop=0.9,
ymin=0)

dcaoutput

dcadf <- data.frame(dcaoutput$net.benefit)

temp <- melt(dcadf, id="threshold",

           measure=c("ANN", "RF", "NB", "Lreg", "Bagging", "Boosting"))

library(ggplot2)

ggplot(temp,

      aes(x=threshold,

         y=value,

         colour=variable,

         group=variable)) + geom_line() +

  geom_line(size=0.6) + # Thicker line

  coord_cartesian(xlim = c(0, 0.175), ylim= c(0, 0.10)) +

  labs(x="Threshold probability (%)") + labs(y="Net benefit") +

  theme_minimal() + theme(legend.position = c(0.8, 0.8),

                      text = element_text(size=12), #text size

                      panel.grid.major = element_blank(), #grid lines

                      panel.grid.minor = element_blank(),
```

```
                    axis.line = element_line(colour = "black"), # solid line
                    axis.ticks = element_line(size = 1), #tick marks
                    panel.border = element_rect(linetype = "solid",
                                              fill = NA, colour = "black"), #axis boarders
                    axis.text = element_text(color = "black")) + #text color
scale_color_manual(values = c("Black", "Blue", "Green", "Red", "Orange",
                       "DarkGray"), #Use same colors as with ROC curve
                labels = c("Artificial neural networks","Random forest",
                       "Naive bayes",
                       "Logistic regression",
                       "Bagging",
                       "Boosting")) + labs(color="")
```
